# Trust as a mechanism of system justification

**DOI:** 10.1371/journal.pone.0205566

**Published:** 2018-10-12

**Authors:** Katarzyna Samson

**Affiliations:** Wroclaw Faculty of Psychology, SWPS University of Social Sciences and Humanities, Wroclaw, Poland; Universidad Loyola Andalucia, SPAIN

## Abstract

People are motivated to hold favorable attitudes about the systems on which they depend, so they justify (i.e., rationalize, defend and bolster) forms of social and economic inequality, even if the inequality is disadvantageous to them. This paper examines how this system-justifying motivation is reflected in behaviors involving interpersonal trust. In a series of three experiments using the trust game I manipulate income inequality by providing participants with higher (advantaged position) or lower (disadvantaged position) initial endowments and measure their trust toward individuals on the same or on different positions. Results show that higher income individuals trust other higher income individuals more than lower income individuals (ingroup favoritism), while lower income individuals trust higher income individuals more than lower income individuals (outgroup favoritism). It is also shown that the strength of these biases is dependent on the level of endorsement of system justifying ideology and the legitimacy of the system. More trust toward those in advantaged positions within a social system, expressed both by equally advantaged as well as by disadvantaged others, not only secures the advantaged in their positions but also reinforces the underlying inequality.

## Introduction

One of the reasons why social, economic and political arrangements based on inequality (i.e., in which some people have more money, power, opportunities or other resources than others) persist, is because people feel a need to justify and support systems in which they function, even ones that are harmful to themselves or their groups [1–2]. Members of disadvantaged groups (e.g., lower status, lower class, racial minorities) endorse the belief that unequal social systems are fair and necessary, sometimes even more than those who are advantaged (e.g., higher status, upper class, Whites). To illustrate it with an example, Trevor Noah, host of The Daily Show, in his autobiography [3] writes about growing up in apartheid South Africa as a child to a Black mother and a White father. He describes an incident when he accidentally hurt his Black cousin’s eardrum during play. When his Black grandmother found out what happened, she beat the Black cousin for it, but not Noah. When explaining why she treated the two kids differently, Noah recalls her saying “I don’t know how to hit a White child… I don’t want to kill a white person. I’m so afraid. I’m not going to touch him” (p. 52). By giving preferential treatment to the non-Black child, the grandmother was justifying and perpetuating a system of inequality in which the Blacks–like herself–were considered less worthy than the Whites.

The breadth of situations in which system justification processes operate is described in terms of Parson’s (1951, p. 25, as cited in [4]) definition of a social system as a structured network of social relations, that is a “system of processes of interaction between actors”. It assumes that there exists some differentiation or clustering of relations between individuals/groups within the social order, such as status, distribution of resources, or division of social roles [4]. Such systems can be relatively tangible, as it is in case of institutions or society, or more abstract, as it is with the unwritten rules of interpersonal behavior. They can also range in size from large-scale systems, such as a nation, to smaller-scale ones, like a family. Although most research done in the system justification theory framework applies to the context of intergroup relations [1–2, 5], it has been shown that legitimation processes are not only elicited in hierarchical intergroup relations, but also in interpersonal, even dyadic ones, such as those involving a supervisor and an employee [6–7].

Most of the work on system justification has been done in the fields of intergroup relations and stereotyping, but the presence of system-justifying motivation was shown in areas as diverse as voting behaviors [8], policemen’s shoot or not decisions [2] or the choice of baby names [9]. In this work I show that system justification motivation is also realized through interpersonal relationships of trust. I adopt a definition of trust as “a psychological state composing the intention to accept vulnerability based on positive expectations of the intentions or behavior of another” ([10] p. 395).

## Crisis of trust

Today’s world is challenged by a crisis of trust. The 2017 Edelman Trust Barometer survey conducted in 28 countries revealed that the general population’s trust in all four key institutions–business, government, NGOs and media–has fallen precipitously and has reached all-time lows in the majority of participating countries [11]. Eurobarometer surveys conducted in 27 European Union countries between 2004 and 2015 also show a rapid decrease in the levels of trust since the 2008 economic crisis [12]. According to the General Social Survey, levels of both interpersonal trust and confidence in societal institutions in the United States have been systematically decreasing since the 1970s [13–14]. The viral nature of the tide of distrust, which commenced among the lower class, spread to the middle class and is now lapping at the feet of the upper class, is seriously alarming [15].

## Trust and economic inequality

The fall in trust has mostly been linked to economic factors, especially the rise of economic inequality. In unequal societies people trust each other less than in more equal communities. A number of studies have shown that higher income inequality is related to lower generalized trust at a national level [14, 16–24].

The most widespread explanation for this relationship is social ties—most individuals are more inclined to trust those who are socially closer and more similar to themselves, including in terms of income and wealth, because familiarity breeds trust [25]. Moreover, social ties mean an increased probability of repeated interaction, which creates incentives for trustworthiness [26]. Societies that are highly stratified are more closed, so it is less likely that people from different strata will meet each other; this results in less trust [18, 27]. Inequality makes people in different strata less likely to have common norms and values [28] or to share a sense of common fate [19] which also reduces their propensity to trust one another. Further, higher inequality could reduce optimism and a sense of having control over one’s life: “where inequality is high, people will be less likely to believe that the future looks bright, and they will have even fewer resources to believe that they are the masters of their own fate” ([19] p. 869).

Other explanations for the negative relationship between trust and economic inequality attribute it mostly to those in disadvantaged positions. Marxists see economic inequality as a signal of exploitation–i.e., untrustworthy behavior–that reduces trust in the top of the social hierarchy [17]. Fischer and Torgler [29] propose that envy affects the perceptions of others’ fairness, which leads to distrust on the part of the disadvantaged under conditions of inequality. Cohen and Steele [30] argue that in unequal societies members of disadvantaged groups show decrements of trust as a result of stigmatization. Sztompka [31] claims that upper class individuals more often believe that they are trusted by others which–given the reciprocal character of trust–leads to greater trust on their part than on the part of the lower class. Also, for the upper class the pain of loss in the case that their trust is betrayed is lower, as they have more resources to fall back on. Yet another link is provided by the opportunity cost of time–for the advantaged, working and trusting is more attractive than spending time verifying the trustworthiness of others, while the disadvantaged cannot afford to take the same risks and are hence more cautious with their trust [17].

Survey data shows that disadvantaged individuals are generally less trusting than the advantaged [11, 16, 23, 32] but experimental research addressing this relationship gives inconsistent results. On the one hand, Korndorfer, Egloff and Schmukle [33] show using large-scale survey data that higher class individuals were more trusting than lower class individuals in a trust game. In the same line, Lount and Pettit [34] show that high status individuals express more trust than low status individuals because of their stronger belief in the others’ benevolent intentions. On the other hand, Piff and colleagues [35] show that lower class individuals are more trusting relative to higher class individuals because of their cooperative and egalitarian values, while Anderson, Mellor and Milyo [36] found no effect of experimentally induced inequality on trust.

When differentiating between high and low status interaction partners, Tropp and colleagues [37] show that members of disadvantaged groups were more more trusting toward lower-status (their ingroup) members than higher-status (outgroup) members, while members of advantaged groups expressed the same levels of trust in an imagined interaction with an outgroup member as with an ingroup member. In contrast, Lei and Vesely [38] show that only the advantaged exhibit an in-group bias in their trusting behaviors, while the disadvantaged trust the advantaged and the disadvantaged to the same extent. Smith [39] shows yet another pattern of results—lower status individuals were more trusting of higher status individuals than lower status individuals, while higher status individuals trusted lower and higher status individuals to the same extent.

These contradicting results suggest that the levels of trust expressed by those in advantaged and disadvantaged positions may not simply derive from the characteristics of one or the other group (such as their social orientation or beliefs about the intentions of others), but rather depend on other variables. I hypothesize that one such variable, which affects the levels of trust among and between those in advantaged and disadvantaged positions, is their relationship with the system responsible for the inequality. This hypothesis is backed by survey data, which shows that during the recent crisis of trust, people believe that overall the system is failing them–they suffer from a sense of injustice, lack hope and confidence, and have an increased desire for change [11].

### System justification theory

In social psychology relationships with the system have been most thoroughly addressed by system justification theory [1–2]. The theory postulates that just as people are motivated to hold favorable attitudes about themselves and the social groups to which they belong, they are also motivated to hold favorable attitudes about the social, economic, and political systems on which they depend. As such systems are usually organized hierarchically, with some individuals or groups controlling others [40], a motivation to justify the system typically leads its members to defend, bolster, and rationalize forms of social and economic inequality, even if the inequality is disadvantageous to them and/or to their ingroup [41–43].

This motivation to justify the system can be directly realized through the endorsement of ideologies that serve to protect, perpetuate, and justify the status quo, such as protestant work ethic, meritocracy, fair market ideology, economic system justification, opposition to equality, belief in a just world, social dominance orientation, right-wing authoritarianism or political conservatism [4, 42]. System justification motivation can also be realized in an indirect way, through the internalization of the existing social order. For the advantaged, it means rationalizing one’s own position of advantage, which leads to expressions of ingroup favoritism. For the disadvantaged, the situation is reversed–rationalizing the status quo means accepting responsibility for, or simply accepting, being disadvantaged, and leads to outgroup favoritism–“the expression of an evaluative preference for members of a group to which one does not belong” [2].

In groups that have traditionally been targets of discrimination and prejudice, an explicit expression of outgroup favoritism is highly unlikely, because of the strength of prescriptive norms to avoid identification with the oppressor [44]. There also exist quite strong normative pressures for members of advantaged groups to avoid being seen as prejudiced or discriminatory [45]. In such cases the internalization of the existing order may be stronger at an unconscious than at a conscious level, insofar as such impulses would be less subject to controlled processing and the activation of conflicting motives [2, 46]. Both direct and indirect manifestations of system justifying motivation are related to one another, which further suggests that they may serve a similar ideological function, namely to legitimize existing social arrangements [42, 47–49]. Evidence summarized by Jost et al. [2] indicates that an acceptance of system justifying ideologies is associated with increased ingroup favoritism among members of different high status groups and with increased outgroup favoritism among members of low status groups.

A number of moderating variables have been shown to affect the strength of system justifying motivation [6, 42, 46, 50]. One is the level of endorsement of system justifying ideologies. Although some of these ideologies focus on the social and cultural, while others concern economic matters, all share similar cognitive and motivational antecedents and produce similar consequences for individuals, groups, and systems–anchoring the status quo and exaggerating the fairness and justifiability of the system [42, 50]. Another moderating variable is system legitimacy, i.e., being in accord with the norms, values, beliefs, practices and procedures accepted by a group [51]. When the system is perceived as more legitimate, the internalization of the existing social order is stronger, and so is the conviction that one lives in a predictable, safe, and supportive environment [2, 52–53]. Conversely, when the system is entirely unpredictable and it is impossible to learn the rules governing it or to develop a sense of efficacy and control over one’s outcomes, although the need for system justification may exist, it is unlikely to be satisfied adequately [54]. This situation is typical of authoritarian regimes [55–56] or societies with a high level of anomie [54]. In such cases perceiving the system as utterly illegitimate can be preferable to perceiving it as legitimate but generating random and capricious outcomes [57]. Therefore, when a system does not adhere to its own rules, its members may choose to treat it as unjustifiable and seek satisfaction of their system justification need elsewhere [54].

### Overview of present research

The aim of this research was to verify if system justifying motivation is reflected in the levels of trust within a social system. In particular, it focused on interpersonal trust between lower and higher income individuals in a system characterized by economic inequality. Preliminary evidence supporting this hypothesis comes from research conducted on a group of Latinos in the US showing that the lower income population declared a greater degree of trust in government officials to do what is right than the higher income group, which points to a system-legitimizing facet of trust [42]. While economic inequality encompasses the distribution of various economic variables such as income, wealth, access to goods or consumption, in this paper I will focus solely on inequality of income, as it can be easily operationalized within adopted methodology.

To test this hypothesis, I examined the effects of income and inequality on trust in a series of experimental studies. Overall I predicted that under conditions of inequality: (a) higher income individuals would express ingroup favoritism, i.e., more trust toward other higher income individuals than toward lower income individuals; while (b) lower income individuals would express outgroup favoritism, i.e., more trust toward higher income individuals than toward their lower income fellows. This basic pattern indicating the presence of a system justifying motivation was demonstrated in Study 1. The following studies focused on moderating variables that affect the strength of a system justifying motivation. In Study 2 I hypothesized that a system justifying motivation expressed through trust would be dependent on the strength of endorsement of the system justifying ideology. I added a measure of economic system justification and demonstrated that system justification is only reflected in trusting behaviors of high system justifiers. In Study 3 I hypothesized that system justifying motivation would be dependent on the legitimacy of the system. Using an additional legitimacy manipulation, I demonstrated that when the system failed to comply with its own rules the pattern of results indicating the presence of a system justifying motivation disappeared.

All research procedures were approved by an ethics committee (Komisja RW ds. Etyki Badań Naukowych) at the Faculty of Psychology, University of Warsaw. All participants gave informed consent prior to participating, verbally in the laboratory studies (Studies 1 and 3) and by clicking an indicated button when the research was conducted online (Study 2). The verbal consent procedure was approved by the ethics committee and was obtained in the presence of both a laboratory assistant who ran the study and the author of this paper. Written consent was not obtained to protect participant anonymity; at the time this was the standard procedure for obtaining informed consent in studies with no follow-up procedures that did not pose any significant threat to the participants’ well-being. All research was carried out in accordance with the committee’s recommendations.

## Study 1

### Method

Study 1 was conducted among 120 (73% female) students aged 18–32 (*M* = 21.64; *SD* = 2.81). Participants received a small remuneration for participation (ca. 1–2 euros in local currency, the exact amount depending on their decisions made in the study).

In the study participants played *the trust game* [58]–an economic game in which two players can increase their wealth through the expression and reciprocity of trust. At the outset of the game, each player receives a set amount of money that constitutes their initial endowment. The first player decides how much of their initial endowment they would like to send to the second player, knowing that the whole transfer will be tripled once the other person receives it. Then, the second player returns any fraction of currently possessed money (i.e., their initial endowment enlarged by the received transfer) to the first player. After both players have made their decisions, a round of the trust game ends. The amounts sent by players are measures of their expression (first player) and reciprocity (second player) of trust. Throughout this research I used the percentage of possessed money sent to the other player as an index of trust.

I experimentally manipulated participants’ income (higher vs. lower) and their standing relative to their partner (equality vs. inequality) within the structure of the game. To manipulate income, I provided participants with different initial endowments to use in the game. They could either receive 5 units of experimental currency (lower income) or 10 units of experimental currency (higher income). Equality vs inequality was manipulated with the participant’s partner’s income. The partner could receive an equal income (equality condition) or an unequal income (inequality condition; when the participant had the lower income, their partner received the higher income and vice versa). Similar manipulations were used by Brulhart & Usunier [59] and Smith [38]. Table 1 summarizes the experimental conditions in Study 1.

**Table 1:** Initial trust game endowments depending on experimental condition (Study 1).

Condition	Participant’s endowment	Partner’s endowment
Inequality / higher income	10	5
Inequality / lower income	5	10
Equality / higher income	10	10
Equality / lower income	5	5

Before the trust game started, participants became acquainted with the rules of the game and drew initial endowment sizes. Participants were told that half of the players receive 5 units of experimental currency and the other half receive 10 units of experimental currency and that these amounts are appointed at random. Given the asymmetry between the role of the first and the second player in the structure of the trust game, I also controlled for player order, so participants were randomly assigned to be either first or second players. The role of the participant’s partner was played by a preprogrammed computer strategy that always sent them half of money it had (initial endowment in the role of the first player; initial endowment enlarged by the received transfer in the role of the second player) to the participant. The game consisted of a single round, but the participants did not know how many rounds of the game they would be playing.

### Results

I performed a two-way univariate ANCOVA with trust as the dependent variable, income and inequality as fixed factors, and controlling for player order. Overall, the model was significant, *F*(4,115) = 17.30, *p* < .001, adjusted *R*^*2*^ = .35. The main effect of income was significant, *F*(1,115) = 8.83, *p* = .004, η_p_^2^ = .07, indicating that overall lower income individuals trusted more (*M* = 50.03, *SD* = 25.94) than higher income individuals (*M* = 39.20, *SD* = 22.65). The main effect of inequality was not statistically significant, *F*(1,115) = 0.18, *p* = .67. The main effect of player order was significant, *F*(1,115) = 47.40, *p* < .001, η_p_^2^ = .29.

**Fig 1:**
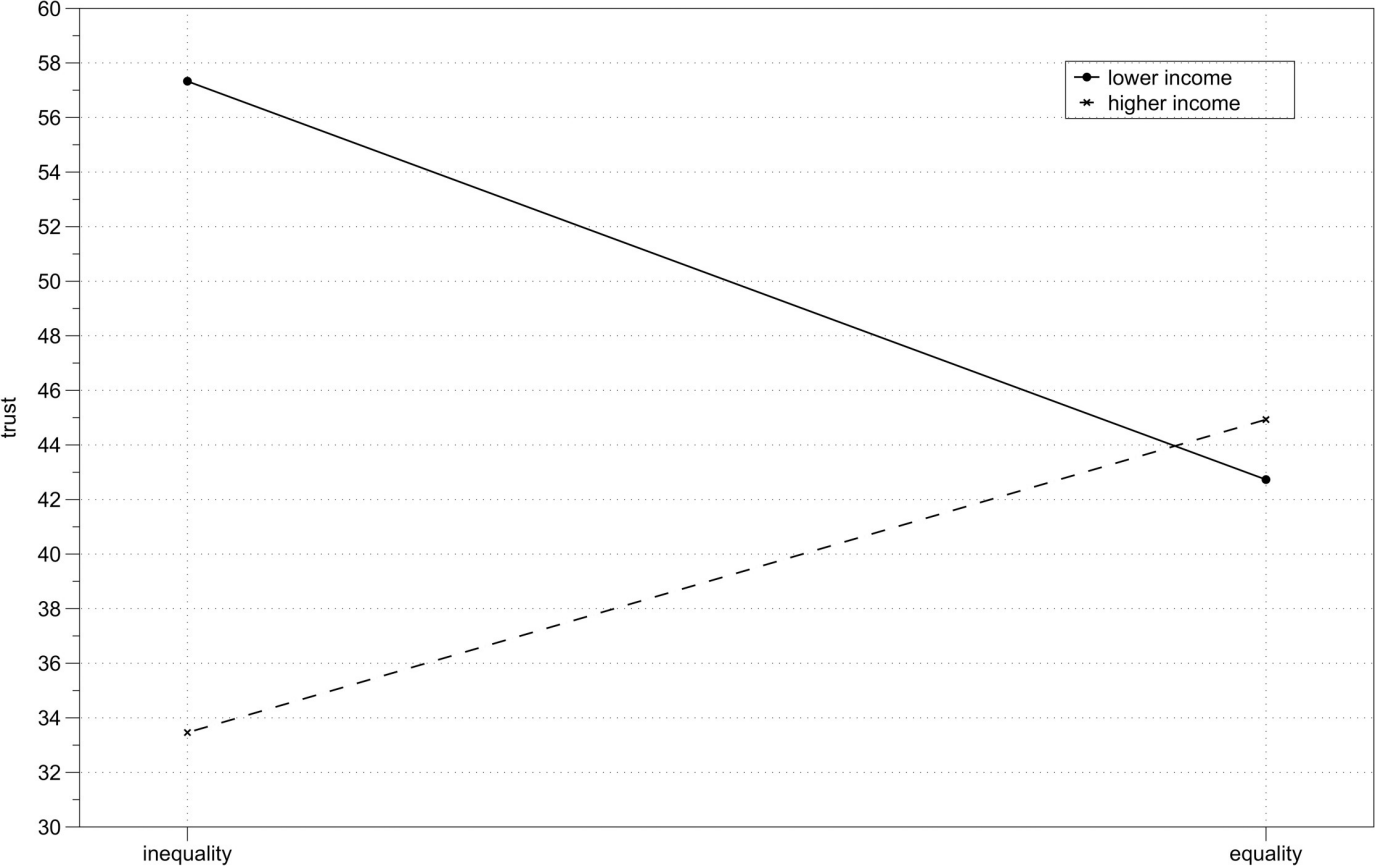
Study 1 results. Mean trust toward unequal and equal income partner depending on participant’s income (higher vs lower).

**Fig 2:**
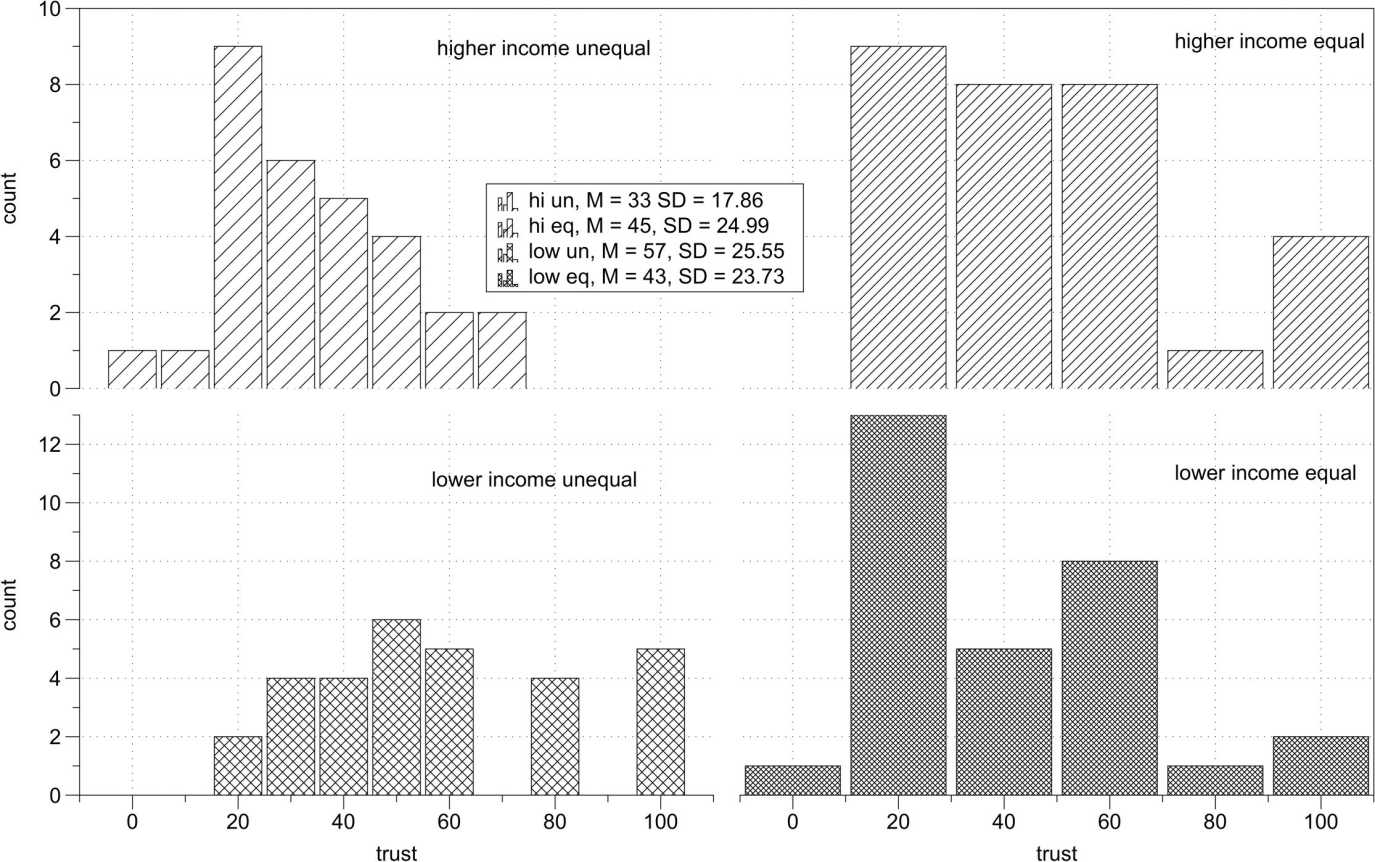
Study 1 results. Distribution of trust scores across experimental conditions.

There was a significant interaction between income and inequality, *F*(1,115) = 12.79, *p* = .001, η_p_^2^ = .10 (Fig 1), so I computed simple main effects with Bonferroni adjustment for multiple comparisons. Among higher income individuals, there was a significant effect of inequality, *F*(1,115) = 4.95, *p* = .03, η_p_^2^ = .04, indicating that they trusted higher income partners more (*M* = 44.93, *SD* = 25.42) than they trusted lower income partners (*M* = 33.46, *SD* = 18.16). Among lower income individuals the effect of inequality was also significant, *F*(1,115) = 8.02, *p* = .005, η_p_^2^ = .07, indicating that lower income individuals trusted higher income partners (*M* = 57.33, *SD* = 25.99) more than lower income partners (*M* = 42.73, *SD* = 24.14). The simple main effect of income was significant in the inequality condition *F*(1,115) = 21.43, *p* < .001, η_p_^2^ = .16, indicating than in the context of unequal relations lower income individuals trusted more than higher income individuals. In the equality condition, the simple main effect of income was not significant, *F*(1,115) = 0.18, *p* = .67, indicating that the levels of trust toward equal income partners were not different for higher income than for lower income individuals. Descriptive statistics of trust in each experimental condition are presented in Table 2. Distribution of trust scores across experimental conditions is presented on Fig 2.

**Table 2:** Descriptive statistics of trust across experimental condition (Study 1).

Condition	N	M	SD
Inequality / higher income	30	33.46	18.16
Inequality / lower income	30	57.33	25.98
Equality / higher income	30	44.93	25.42
Equality / lower income	30	42.73	24.14
Total	120	44.61	24.85

### Discussion

The aim of the first study was to verify the presence of intergroup biases indicating the presence of system justifying motivation in interpersonal relationships of trust–ingroup favoritism among higher income individuals and outgroup favoritism among lower income individuals. The results confirmed both the predicted effects. Higher income individuals expressed and reciprocated more trust toward other individuals with higher incomes than toward those with lower incomes. Lower income individuals expressed and reciprocated more trust toward higher income individuals than toward fellow lower income individuals.

Based on these results only, one could attribute the difference in average levels of trust among higher and lower income individuals to the difference in their initial endowments (along the lines of “the more you have, the less trusting you are”, i.e., stake size effect) and not to the experimental effect of inequality manipulation. But the results also showed that average trust expressed / reciprocated by both higher and lower income individuals toward an equal income partner did not differ. This result is in line with previous research–a meta-analysis of trust games shows that as long as both players’ initial endowments are equal, their size does not influence behaviors in a trust game [60]. As the difference between the higher and lower income individuals’ behavior cannot be attributed to the size of their initial endowments alone, it thus must be due to the inequality between them and their trust game partners.

One more significant effect was that of player order–percentagewise, first players expressed more trust than second players reciprocated. This effect stems from the asymmetrical structure of the game–the ratio of money sent to money possessed has a different denominator for first (initial endowment) and second (initial endowment + first player’s transfer) players. A meta-analysis of trust games shows that when players receive equal endowments trustors on average send about 50% of their endowment to the trustees, while the trustees on average reciprocate with about 37% of the money they have [60], so absolute values of trust expression and trust reciprocity from a trust game cannot be directly compared. However, there was no significant interaction effect including player order, which means that when it comes to relationships of trust under conditions of inequality, the general rules governing the behavior of first and second trust game players are the same. This result confirms the assumption that the expression and reciprocity of trust both serve the same system justifying needs.

## Study 2

### Method

Study 2 was conducted on a sample of 200 (48.5% female, aged 16–42, *M* = 25.36, *SD* = 6.05) members of a Polish online research panel Ariadna. Credit received for participation in the study (points exchangeable for goods and/or services) was independent of the decisions made in the study.

At the beginning of the study participants were asked to fill out a measure of economic system justification. Then they played the trust game as in Study 1, except for two differences: (a) possible initial endowments were 10 (lower income) and 20 (higher income) units of experimental currency; (b) in the role of the first player the computer entrusted the participant with all of its initial endowment, while in the role of second player it returned 50% of what it had at that moment. Table 3 summarizes the experimental conditions in Study 2.

**Table 3:** Initial trust game endowments depending on experimental condition (Study 2).

Condition	Participant’s endowment	Partner’s endowment
Inequality / higher income	20	10
Inequality / lower income	10	20
Equality / higher income	20	20
Equality / lower income	10	10

**Fig 3:**
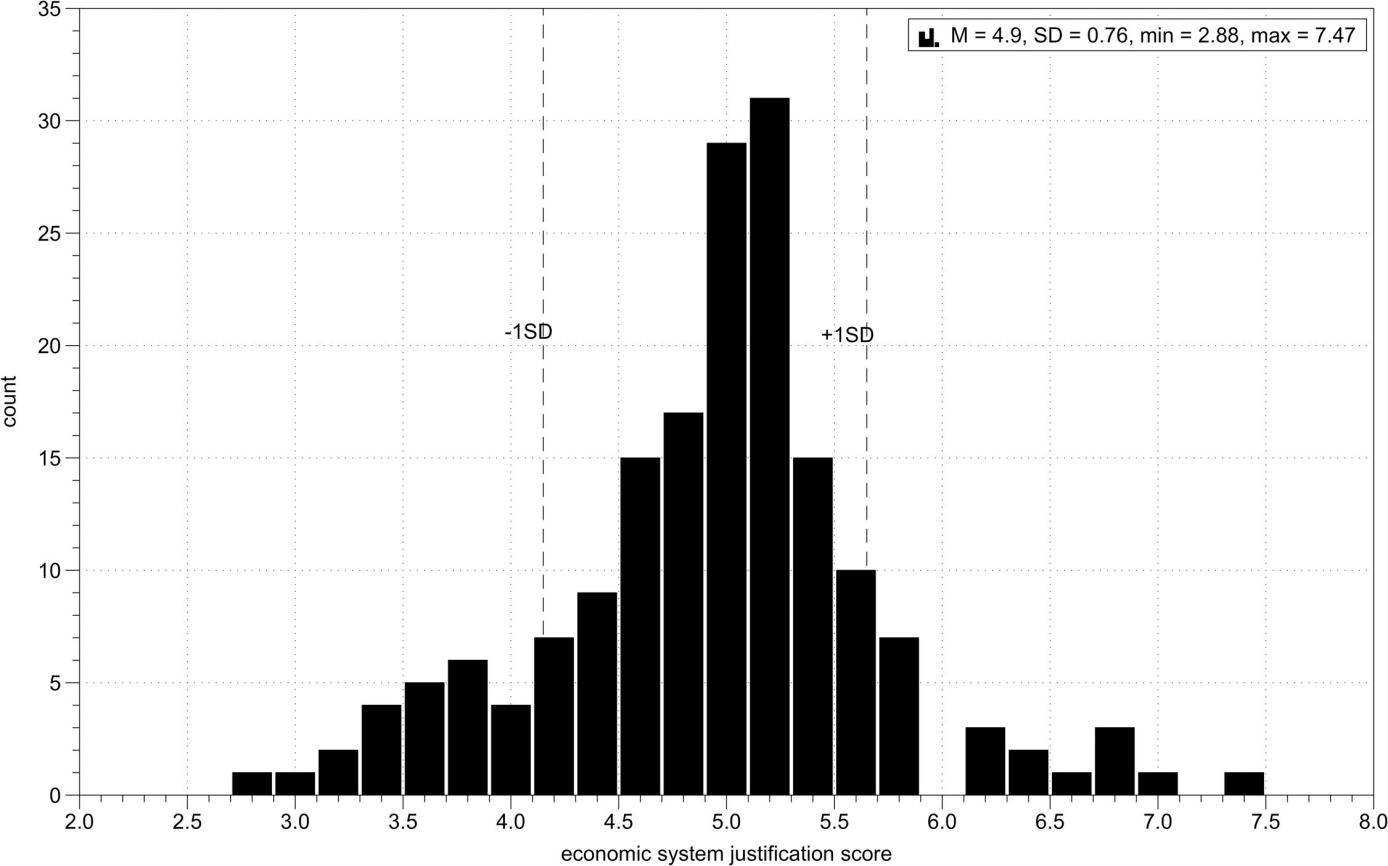
Study 2. Distribution of economic system justification scores.

Economic system justification (α = .62; *M* = 4.93, *SD* = 0.76) was measured with a polish translation of the Economic System Justification scale [47]. Participants were asked to rate on a scale from 1 = completely disagree to 9 = completely agree to what extent they agreed with the following statements indicating a general ideological tendency to legitimize economic inequality (questions 2, 4, 6 8, 10, 13 15 and 17 are reverse-coded): (1) If people work hard, they almost always get what they want. (2) The existence of widespread economic difference does not mean that they are inevitable. (3) Laws of nature are responsible for differences in wealth in society. (4) There are many reasons to think that the economic system is unfair. (5) It is virtually impossible to eliminate poverty. (6) Poor people are not essentially different from rich people. (7) Most people who don't get ahead in our society should not blame the system; they have only themselves to blame. (8) Equal distribution of resources is a possibility for our society. (9) Social class differences reflect differences in the natural order of things. (10) Economic differences in the society reflect an illegitimate distribution of resources. (11) There will always be poor people, because there will never be enough jobs for everybody. (12) Economic positions are legitimate reflections of people's achievements. (13) If people wanted to change the economic system to make things equal, they could. (14) Equal distribution of resources is unnatural. (15) It is unfair to have an economic system which produces extreme wealth and extreme poverty at the same time. (16) There is not point in trying to make incomes more equal. (17) There are no inherent differences between rich and poor; it is purely a matter of the circumstances into which you are born. The distribution of economic system justification scores is shown on Fig 3.

The study was conducted online, so I added comprehension checks after the game to ensure that participants had followed the procedure. Participants were asked to indicate (a) how the initial endowments were distributed in the game (equally or if they received more/less than their partners); and (b) which of a list of possible strategies best describes the strategy they used in the game, (e.g. *I played to win the most for myself* or *I didn’t understand the rules of the game*). Participants who failed to identify how initial endowments in the game were distributed (n = 16) or indicated they didn’t understand the rules of the game (n = 9) were excluded from the analyses. As the trust game is a tool of behavioral economics, one participant who identified himself as an economist was also excluded. The final sample consisted of 174 participants (48.9% female), aged 16–42 (*M* = 25.18, *SD* = 5.81).

### Results

I performed a two-way univariate ANCOVA with trust as the dependent variable, income and inequality as fixed factors, controlling for player order. Overall, the model was significant, *F*(4,169) = 3.55, *p* = .008, adjusted *R*^*2*^ = .06. The main effects of income and inequality were not statistically significant, *F*(1,169) = 1.48, *p* = .23 and *F*(1,169) = 0.81, *p* = .37, respectively. Main effect of player order was significant, *F*(1,169) = 5.95, *p* = .02, η_p_^2^ = .03.

**Fig 4:**
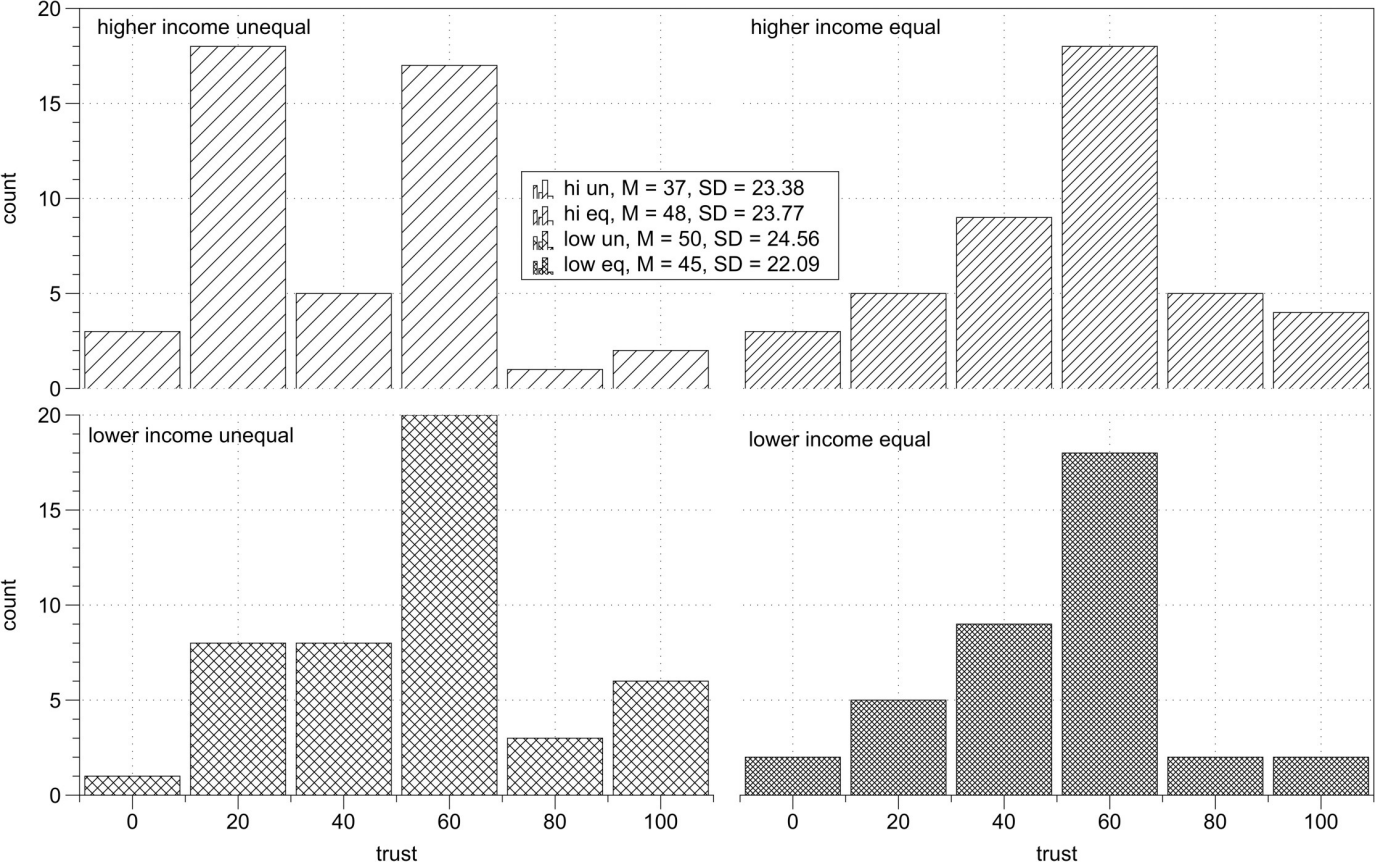
Study 2 results. Distribution of trust scores across experimental conditions.

There was a significant interaction between income and inequality, *F*(1,169) = 6.07, *p* = .02, η_p_^2^ = .04, so I computed simple main effects with Bonferroni adjustment for multiple comparisons. Among higher income participants, there was a significant effect of inequality, *F*(1,169) = 5.87, *p* = .02, η_p_^2^ = .03, indicating that higher income participants trusted partners with equally higher income more (*M* = 48.21, *SD* = 24.05) than than they trusted lower income partners (*M* = 36.98, *SD* = 23.64). Among lower income participants the effect of inequality was not significant, *F*(1,169) = 1.19, *p* = .28, they trusted higher income partners (*M* = 49.94, *SD* = 24.83) as much as fellow lower income partners (*M* = 44.61, *SD* = 22.39). Simple main effects of income were significant in inequality conditions *F*(1,169) = 7.22, *p* = .008, η_p_^2^ = .04, indicating that in the context of unequal relations lower income participants trusted higher income participants more than higher income participants trusted lower income participants. In the context of equal relations, simple main effect of status was not significant, *F*(1,169) = 0.73, *p* = .39, indicating that the levels of trust toward equal income partners did not differ between lower and higher income participants. Descriptive statistics of trust in each experimental condition are presented in Table 4. Distribution of trust scores across experimental conditions is presented on Fig 4.

**Table 4:** Descriptive statistics of trust across experimental condition (Study 2).

Condition	N	M	SD
Inequality / higher income	46	36.98	23.64
Inequality / lower income	46	49.94	24.83
Equality / higher income	44	48.21	24.05
Equality / lower income	38	44.61	22.39
Total	174	44.91	24.14

I also tested for the moderating role of economic system justification by conducting a regression analysis with income and inequality as predictors, economic system justification as a moderator and player order as a covariate. Overall the model was significant, *F*(8,165) = 2.62, *p* = .01, adjusted *R*^*2*^ = .07, all regression coefficients are presented in Table 5.

**Table 5:** Summary of regression analysis of trust with income and inequality as predictors, economic system justification as a moderator and player order as a covariate.

Predictor	*B(SE)*	B
Income	13.03 (12.19)	0.54
Inequality	22.60 (12.21)	0.94†
Player order	-7.02 (2.62)	-0.15†
ESJ	2.10 (2.46)	0.07
Income x Inequality	-15.93 (12.17)	-0.66
Income x ESJ	-3.04 (2.45)	-0.63
Inequality x ESJ	-4.24 (2.46)	-0.88†
Income x Inequality x ESJ	4.11 (2.45)	0.85†
*F*	2.62**	
Adjusted *R*^*2*^	.07	

Experimental conditions were effect coded (income: -1 = lower, 1 = higher; inequality: -1 = unequal, 1 = equal).

† p < .10.

** p < .01.

**Fig 5:**
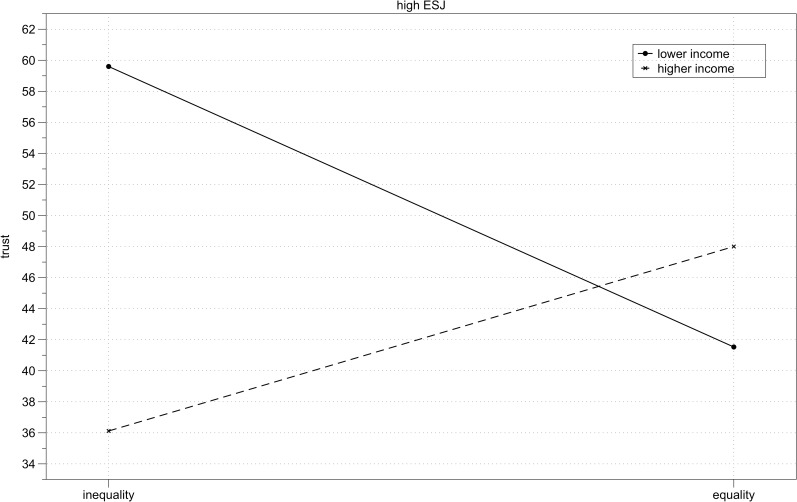
Study 2 results–high system justifiers. Mean trust toward unequal and equal income partner depending on participant’s income (lower vs higher) for participants with high scores on the economic system justification measure.

**Fig 6:**
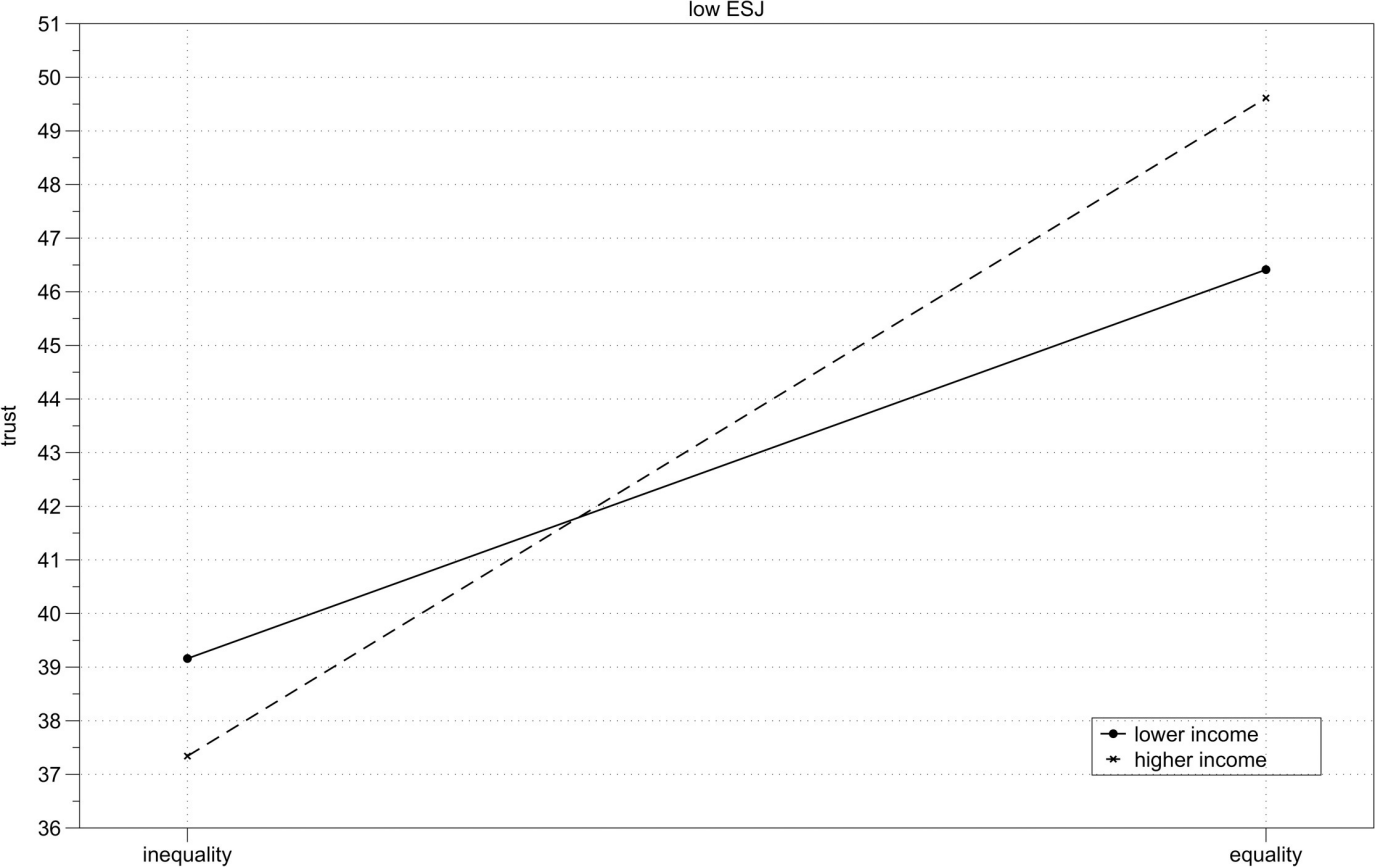
Study 2 results–low system justifiers. Mean trust toward unequal and equal income partner depending on participant’s income (lower vs higher) for participants with low scores on the economic system justification measure.

The hypothesized three-way interaction between income, inequality and economic system justification was marginally significant, *B* = 4.11, *SE* = 2.45, *p* = .09. A simple slopes analysis conducted using PROCESS [61] showed that the interaction between income and inequality was significant for participants with high (+1SD) scores on the economic system justification measure (*B* = 7.49, *SE* = 2.60, *p* = .01), but not significant for participants with low (-1SD) scores on the economic system justification measure, *B* = 1.25, *SE* = 2.55, *p* = .62 (Figs 5 and 6). I further inspected the significant interaction for high economic system justification. Analysis of simple slopes showed that among high economic system justifiers, lower income participants trusted unequal income partners (i.e., higher income) more than they trusted equal income partners, *B* = -9.03, *SE* = 4.02, *p* = .03, while higher income participants trusted equal income partners more than they trusted unequal income partners (i.e., lower income), *B* = 5.94, *SE* = 3.33, *p* = .08. Under conditions of income inequality lower income participants expressed more trust than the higher income participants, *B* = -11.73, *SE* = 3.63, *p* = .001, but when their interaction partners had equal income, the levels of trust did not differ between lower and higher income participants, *B* = 3.23, *SE* = 3.75, *p* = .39.

### Discussion

When I first examined system justifying intergroup biases among lower and higher income participants in the whole sample (as in Study 1), only ingroup favoritism in the higher income group was confirmed. However, when I compared individuals with low and high scores on the economic system justification measure, it turned out that both intergroup biases indicating system justifying motivation (ingroup favoritism among higher income participants and outgroup favoritism among lower income participants) were present among high economic system justifiers. Low system justifiers were less trusting under conditions of inequality than in case of equality, independently of their own income.

## Study 3

### Method

Study 3 was conducted among 120 (71.6% female) students aged 18–27 (*M* = 21.38, *SD* = 2.22). Participants received remuneration for participation, as in Study 1.

The experimental procedure in Study 3 was similar to that in previous studies, but rather than assigning participants to higher or lower income conditions at random, their position was manipulated with an introductory task. At the beginning of the study participants were informed that it consisted of two parts–an introductory task and a game–and that the aim of the introductory task (writing down as many different uses for a box of matches as possible in 3 minutes) was to assign the players’ starting positions in the game. Participants were told that the person who performed better in the introductory task would get a higher initial endowment in the game (higher income, 10 units of experimental currency) and the person who performed worse would get a lower initial endowment (lower income, 5 units of experimental currency).

In the legitimate inequality conditions participants were either: (a) told they performed better in the introductory task than their partner (ostensibly in another laboratory) and were given a higher initial endowment in the game, in line with presented rules (legitimate / higher income condition); or (b) told they performed worse in the introductory task and were given a lower initial endowment in the game, in line with presented rules (legitimate / lower income condition). In the illegitimate inequality conditions participants’ initial endowments in the trust game contradicted the results of the introductory task and procedural instructions presented by the experimenter. Participants were either: (c) told they performed worse in the introductory task but were given a higher initial endowment in the game (illegitimate / higher income condition); or (d) told they performed better in the introductory task but were given a lower initial endowment in the game (illegitimate / lower income condition). Unlike in previous studies, in this study there were no equality conditions. Table 6 summarizes the experimental conditions in Study 3.

**Table 6:** Introductory task feedback and initial trust game endowments depending on experimental condition (Study 3).

Condition	Introductory task feedback	Participant’s endowment	Partner’s endowment
Legitimate / higher income	Better than partner	10	5
Legitimate / lower income	Worse than partner	5	10
Illegitimate / higher income	Worse than partner	10	5
Illegitimate / lower income	Better than partner	5	10

After the introductory task participants played a single round of the trust game and filled out the same comprehension checks as in Study 2. All participants correctly identified if they had received a smaller or larger initial endowment in the game and none claimed not to have understood the rules of the game. I also added a manipulation check (*In your opinion*, *how legitimate was the inequality between the players’ initial endowments*?*)* answered on a Likert-type scale (1 = not at all legitimate, 7 = fully legitimate) to verify the efficacy of the legitimacy manipulation.

The new procedure was tested in a pilot study, in which participants (*N* = 22) were subject to the same manipulation as in Study 3, except that there was not only inequality, but also equality conditions (like in Studies 1 and 2). After being informed about the distribution of initial endowments in the trust game they skipped the game and only answered the manipulation check question. The results yielded a marginally significant interaction between legitimacy and inequality, *F*(1,14) = 4.03, *p* = .06, η_p_^2^ = .22. Analysis of simple effects indicated that perception of legitimacy of the distribution of initial endowments is indeed higher under conditions of legitimate inequality, *F*(1,14) = 6.95, *p* = .02, η_p_^2^ = .33, and that when partners’ incomes are equal, their perception of legitimacy does not differ between legitimate and illegitimate conditions, *F*(1,14) = 0.21, *p* = .65. It hence did not make sense to include equal income conditions in Study 3, as it turned out that in an experimental setting the effect of equality overshadowed that of illegitimacy, and participants failed to notice the latter.

### Results

In the legitimate condition, inequality of participants’ initial endowments in the trust game was perceived as significantly more legitimate (*M = 4*.*93*, *SD = 1*.*69*) than in the illegitimate condition (*M* = 3.27, *SD* = 1.64), *t*(118) = 5.50, *p* < .001.

I performed a two-way univariate ANCOVA with trust as the dependent variable, income and legitimacy as fixed factors, controlling for player order. Overall, the model was significant, *F*(4,115) = 20.79, *p* < .001, adjusted *R*^*2*^ = .40. The main effect of income was marginally significant, *F*(1,115) = 3.22, p = .08, indicating that lower income participants trusted more (*M* = 56.53, *SD* = 2.95) than higher income participants (*M* = 49.06, *SD* = 2.95). The main effect of legitimacy was not statistically significant, *F*(1,115) = 0.50, *p* = .48. The main effect of player order was significant, *F*(1,115) = 73.70, *p* = .02, η_p_^2^ = .03.

**Fig 7:**
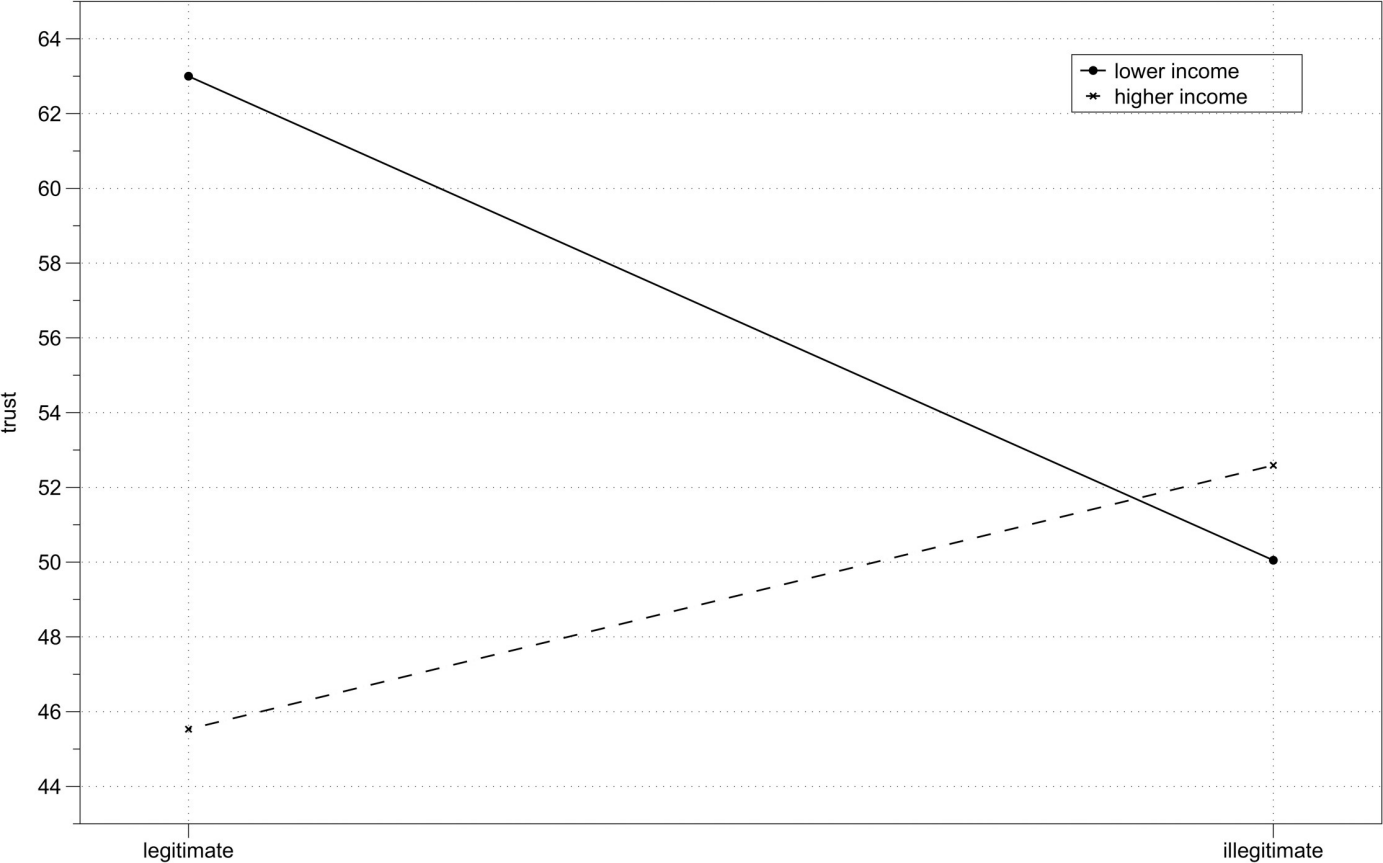
Study 3 results. Mean trust in legitimate and illegitimate inequality conditions depending on participant’s income (lower vs higher).

**Fig 8:**
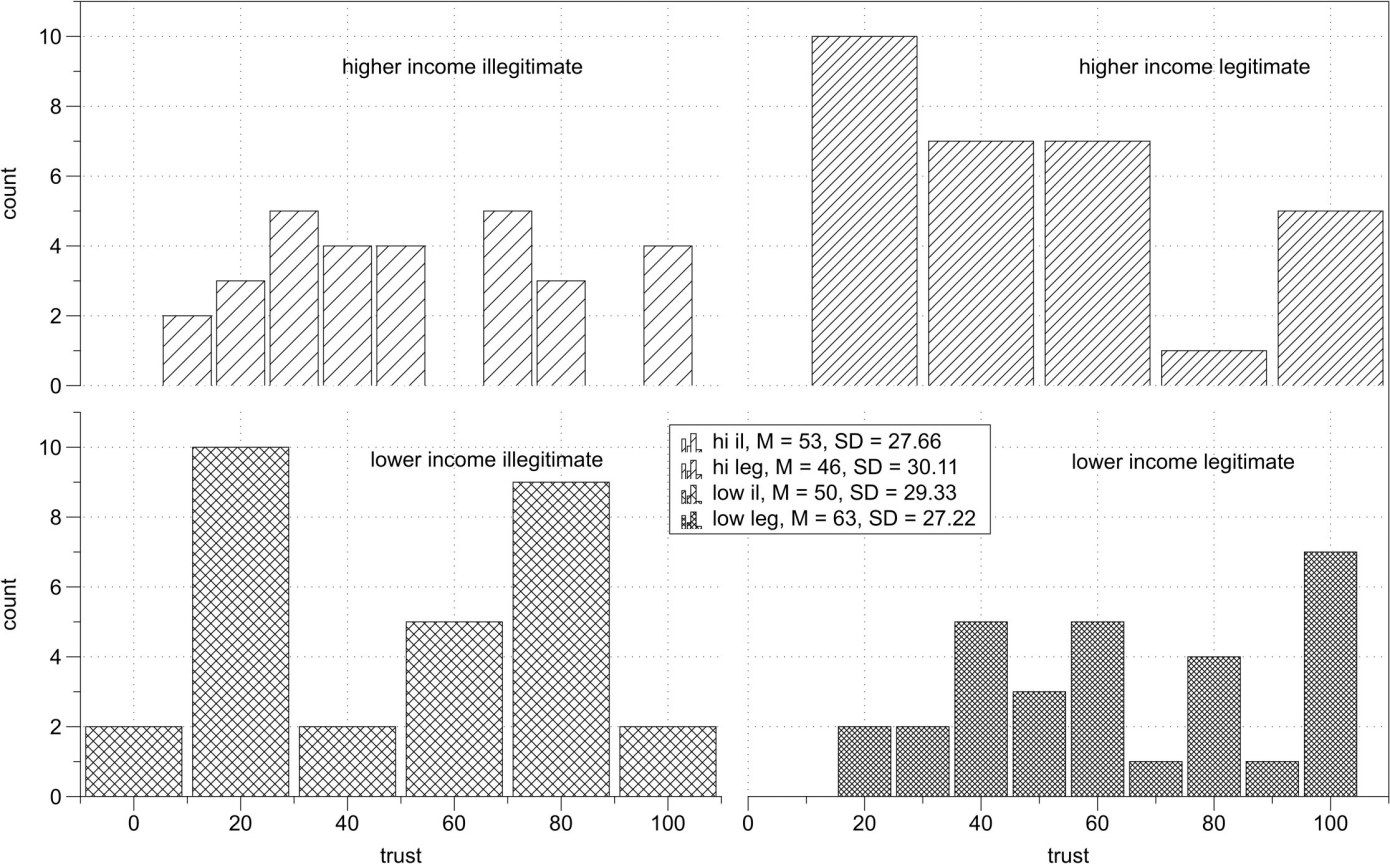
Study 3 results. Distribution of trust scores across experimental conditions.

There was a significant interaction between income and legitimacy, *F*(1,115) = 5.77, *p* = .02, η_p_^2^ = .05 (Fig 7). I computed simple main effects with Bonferroni adjustment for multiple comparisons. Among lower income participants, there was a significant effect of legitimacy, *F*(1,115) = 4.83, *p* = .03, η_p_^2^ = .04, indicating that they trusted higher income partners more under conditions of legitimate (*M* = 63.00, *SD* = 27.69) than illegitimate inequality (*M* = 50.05, *SD* = 29.84). Among higher income participants the effect of legitimacy was not significant, *F*(1,115) = 1.44, *p* = .23, indicating that higher income participants trusted their lower income partners to the same extent under conditions of legitimate (*M* = 45.53, *SD* = 30.62) and illegitimate (*M* = 52.59, *SD* = 28.13) inequality. The simple main effect of income was significant in the case of legitimate inequality, *F*(1,115) = 8.80, *p* = .004, η_p_^2^ = .07, indicating that lower income participants trusted more than higher income participants. In the case of illegitimate inequality, simple main effect of income was not significant, *F*(1,115) = 0.19, *p* = .67. Descriptive statistics of trust in each experimental condition are presented in Table 7. Distribution of trust scores across experimental conditions is presented on Fig 8.

**Table 7:** Descriptive statistics of trust across experimental condition (Study 3).

Condition	N	M	SD
Legitimate / higher income	30	45.53	30.62
Legitimate / lower income	30	63.00	27.69
Illegitimate / higher income	30	52.59	28.13
Illegitimate / lower income	30	50.05	29.84
Total	120	52.79	29.44

### Discussion

The aim of this study was to verify that system justifying motivation as manifest in trusting behavior is moderated by the legitimacy of the system. I expected the system justifying pattern of behavior (more trust on the part of lower income participants toward higher income participants than vice versa) to only be present when the inequality between trust game partners was perceived as legitimate. The results confirmed this hypothesis. When inequality was illegitimate, there were no differences between lower and higher income participants’ behavior.

It is worth noting that the simple effects of legitimacy were significant among lower income participants, but not among higher income participants. This means that lower income participants were more affected by the system legitimacy manipulation. Finding themselves in their disadvantaged positions rightfully (i.e., in the legitimate inequality condition) they justified the system and expressed a lot of trust toward their higher income partners. However, when they were supposed to be in advantaged positions and ended up disadvantaged (i.e., in the illegitimate inequality condition), they did not justify the system and hence did not trust their higher income partners as much. For higher income participants it did not make a significant difference if their advantaged position was achieved rightfully (i.e., in the legitimate inequality condition) or unrightfully (i.e., in the illegitimate inequality condition), in both cases they expressed equal levels of trust toward their lower income partners. This result is in line with system justification theory–while the advantaged (i.e., higher income participants) have no problem reconciling the desire to see the system as fair with the desire to see themselves and their group members in favorable terms, for the disadvantaged (i.e., lower income participants) acceptance of system justifying beliefs entails conflictive needs to justify the status quo and to enhance their own self-esteem and group status [2, 48, 52, 62]. Consequently, when given a good reason to reject a system responsible for inequality, e.g., when it does not adhere to its own rules, those in disadvantaged positions are the first to do so.

## General discussion

The presented results fully confirm the validity of applying a system justification framework to the analysis of relationships of trust. Under conditions of inequality, higher income participants express more trust toward their ingroup, while the lower income participants express more trust toward the outgroup (Study 1). As predicted by system justification theory, these effects are present among high economic system justifiers, but not among low economic system justifiers (Study 2) and only when the system is legitimate (Study 3). System justifying motivations are also more robust among higher income participants than in case of lower income participants. This difference is presumably due to the fact that while for those in advantaged positions system justifying and ego justifying motivations align, for those in disadvantaged positions system justifying motivations counter the motivation to hold favorable attitudes about themselves and their own groups. Hence, they are on average less likely than those in advantaged positions to see the existing system as fair and legitimate [4, 63–64]. Interestingly, in all three presented studies the levels of behavioral trust was unrelated to individuals’ generalized trust attitudes measured using the General Social Survey question before the trust game (Study 1 *r*(119) = -.01, *p* = .88; Study 2 *r*(174) = -.02, *p* = .80, Study 3, *r*(119) = .02, *p* = .81).

A lack of distinction between trust expressed and reciprocated in the game, uncommon in other research employing the trust game, stems from the assumption that they serve the same system justifying function. The expression of trust, and likewise its reciprocation, may either imply an assumption about the partner’s virtues, such as trustworthiness, honesty, or benevolence, or reflect the result of a strategic calculation of potential gains and losses. But independently of the underlying rationale–whether it reflects a moral belief or an economic judgment–more trusting and trustworthy behaviors directed at those in advantaged positions help maintain their dominant position in the system and perpetuate the unequal status quo. This assumption–that the expression and reciprocation of trust are two sides of the same coin–was confirmed by the results of all three studies. In none of them did player order interact with any other variable in the model.

It is also interesting to link these results with the research on the relationship between income and generosity. Having a higher income has been linked to less moral and more selfish behaviors [35, 65–66]. However, Cote and collaborators [67] show that economic inequality moderates the income–generosity relationship. They claim that when income is amassed in the hands of a small proportion of the population, based on the favorable outcomes of social comparisons with the general population, higher income individuals acquire a sense of entitlement–a conviction that they are more worthy than others. This sense of entitlement leads to a belief that the resources rightfully belong to them which, in turn, reduces the higher income population’s generosity. In contrast, when economic inequality is low, higher income individuals can be equally or even more generous than lower income individuals, as their privileged situation makes giving more affordable to them. The results of this project support the idea that economic inequality moderates the relationship between income and prosocial behaviors. In the equality conditions there is no association between participants’ income and their trust game behavior, but under conditions of inequality that relationship becomes negative.

It must also be noted that all the studies were conducted in Poland which, due to its history of communism, provides a rather specific research context. While system justification possesses similar social, cognitive, and motivational antecedents and consequences in Western Capitalist and post-Communist societies, the overall level of system justification is lower in post-Communist societies [54, 68]. Still, system justifying trusting behaviors were observed among both students and online participants. Replicating the results in a capitalist context would undoubtedly shed more light on the investigated phenomena.

It thus seems that as a mechanism of system justification, trust contributes to sanctioning and sustaining social inequality, with all of its negative consequences for the members of disadvantaged groups, such as depressed entitlement, decreased self-esteem, or increased neuroticism [48], undermining the general support for the redistribution of resources and the willingness to help the disadvantaged [69]. However, in the short run it makes the members of a hierarchical system feel better about their situation, regardless of what that situation may be [53]. From this perspective, by regulating the expression and reciprocation of trust, people address some of their most fundamental human needs: epistemic needs to attain certainty and meaning, existential needs to reduce threat and distress, as well as relational needs to manage social relations and achieve shared reality with others [70]. This perspective fits well with one of the classical sociological approaches, in which trust is defined as a set of “socially learned and socially confirmed expectations that people have of each other, of the organizations and institutions in which they live, and of the natural and moral social orders that set the fundamental understandings for their lives” ([71] pp. 164–165).

It is interesting to link the results of this project to Bourdieu’s [72] account of social capital. While most theories of social capital picture it essentially as a heartwarming network of social connections, Bourdieu uses it to explain the cold reality of social inequalities [73]. He defines social capital as “the aggregate of the actual or potential resources which are linked to possession of a durable network of more or less institutionalized relationships of mutual acquaintance and recognition” ([73] p. 249), but unlike others who treat social capital as a resource that works to the advantage of individuals (e.g. [74–77]) Bourdieu puts the emphasis on its power function, i.e., the way in which social relations increase an individual’s ability to advance his or her interests. In this way, social capital is the means through which privileged individuals maintain their position using their connections to privileged others, and the disadvantaged are excluded from sharing the benefits. This link provides a further confirmation of the validity of looking at relationships of trust as a mechanism of sustaining social inequalities. Trust, just like Bourdieusian social capital, constitutes yet another tool in the armory of the elite, deployed to ensure that the status quo is perpetuated.
